# Bibliometric analysis of research trends on factors affecting older adults with mild cognitive impairment

**DOI:** 10.3389/fneur.2024.1440784

**Published:** 2024-10-02

**Authors:** Lei Yang, Rushi Yang, Bo Wang, Tiantian Liu, Ziyi Wang

**Affiliations:** School of Nursing, Xinxiang Medical University, Xinxiang, China

**Keywords:** mild cognitive impairment, elderly, influencing factors, bibliometrics, visual analysis

## Abstract

**Background:**

In recent years, the problem of cognitive impairment in the elderly has become increasingly prominent. Understanding the research trend of influencing factors of mild cognitive impairment, and provide reference for medical staff to early screening of the elderly with mild cognitive impairment.

**Objective:**

Through the visual analysis of the influence factors of the elderly with mild cognitive impairment, the current research status was discussed.

**Methods:**

The relevant literature in the field of influencing factors of mild cognitive impairment in the elderly included in the Web of Science core collection database from 2013 to 2022 was searched. Using software such as Cite Space and VOS viewer to visually analyze literature citations, country, keywords, and development trends.

**Results:**

A total of 547 relevant literatures were included, and the number of publications showed an increasing trend in the past ten years. The United States ranked first in both the number of published papers (157) and centrality (0.34), and the United States and China had a greater influence on the influencing factors of mild cognitive impairment. Alzheimer’s disease, cognitive decline, the elderly, risk factors, are the research hotspot in this field.

**Conclusion:**

Cognitive decline will affect the autonomy of the elderly. Cognitive frailty, MRI is the forefront of MCI research, to understand the research hotspots and frontiers in this field, to conduct early screening and intervention guidance for people with mild cognitive impairment, so as to delay the occurrence of Alzheimer’s disease, and reduce the pressure on family caregivers and society.

## 1 Introduction

In the context of global aging, population aging has become a serious problem for countries around the world. According to the World Health Organization, Alzheimer’s disease is the seventh leading cause of death in the world. It is a degenerative disease of the central nervous system with an insidious onset and progressive progression (1). A recent cross-sectional study shows that there are 15.07 million cases of dementia in people over 60 years of age in China, including 9.83 million cases of Alzheimer’s disease, the prevalence of MCI in people over 60 years of age is 15.5%, and the number of people with mild cognitive impairment reaches 38.77 million, and the incidence rate is constantly rising (2). At present, there is no specific drug for the treatment of AD, and the basic research and clinical research focus more on the pre-clinical stage of dementia, that is, the stage of mild cognitive impairment. Mild cognitive impairment is a transitional state between normal aging and dementia, a clinical syndrome in which cognitive or memory impairment is present but the ability to perform daily life is normal (3). Mild cognitive impairment not only jeopardizes the mental and physical health of older persons, but also imposes a more serious socio-economic burden. It is estimated that by 2030, the cost of mild cognitive impairment in China will reach 2.54 trillion US dollars (4).

Each year, roughly 5–10% of patients with mild cognitive impairment will develop dementia. Understanding the influencing factors of MCI is beneficial for the early identification and intervention of MCI, and slows down the progression of MCI to AD (5). Among the socio-demographic factors, studies have shown that gender, age and education are among the factors affecting cognitive functioning in older adults. Zhang found that being male and having an education level of junior high school and above were protective factors for MCI, while age 70 and above was a risk factor for MCI (6). Similarly, while economic level has a direct impact on cognitive functioning in older adults, economic level can also indirectly affect cognitive functioning by influencing factors such as social support. Lu′s study found differences in cognitive functioning and social support across economic income groups, more pronounced in higher income groups (7). In lifestyle, there are also associations between different dietary patterns and cognitive function, and some studies have found that n-3 PUFA supplementation has a positive effect on cognitive performance in older adults (8). The Araya-Quintanilla F study found no effective results of short-term n-3 polyunsaturated fatty acid supplementation on cognitive performance in Alzheimer’s patients (9).

At present, more and more researchers pay more and more attention to MCI, but there are still many problems in the field of MCI. Due to the lack of clear and systematic diagnostic criteria, the assessment of cognitive function by medical workers will be biased, and the prevalence of cognitive impairment in the elderly in different regions is affected by gender, age, education level and so on (10). The development of mild cognitive impairment has been explored and researched in various fields of study, including biology, physiology, and psychology. However, there is a lack of systematic review on the factors affecting mild cognitive impairment. This study analyzed the field of MCI by searching the Web of Science database and using bibliometric software. The current status of research, research hotspots and frontiers in the field internationally were demonstrated using visualization to provide a reference for in-depth research on mild cognitive impairment in China.

## 2 Materials and methods

### 2.1 Data sources

A ten-year search of the Web of Science Core Collection for literature published between January 1, 2013, and December 31, 2022, on relevant influencing factors in the mild cognitive impairment population of community-dwelling older adults. The data retrieval strategy is as follows: TS = (“mild Cognitive Impairment*” or “mild cognitive disorder*” or “MCI”) AND (“elderly” or “aged”) AND (“influence factor*” or “risk factor*”). The inclusion criteria for cognitive impairment in this study include amnestic cognitive impairment and non-amnestic cognitive impairment. Literature type was set to article, online publications, conference proceedings and conference abstracts were excluded, and English was selected as the language type, resulting in 896 bibliographic records. In order to obtain more accurate analysis results, after reading the literature titles and abstracts, two researchers independently screened and discussed, manually excluding literature unrelated to mild cognitive impairment. Finally, the remaining 547 documents were recorded. The title, year of publication, country or region, institution, journal, references and keywords of each bibliographic record were collected as basic data.

### 2.2 Statistical methods

This study using the https://bibliometric.com/websitedescribes the number of publications in different countries or regions. VOS viewer software generates easy-to-understand bibliometric analysis mapping (11). The screened literature was imported into Cite Space 6.2.R2 visual analysis software in plain text format. Set the time span as 2013 to 2022, time slice as 1, and node type as institution, journal, keyword, and reference for visualization and analysis (12, 13). To obtain a clear view of the network, the clipping approach selects Pathfinder and Pruning sliced networks to eliminate the less visually appealing networks and nodes. Other settings keep the default algorithm.

## 3 Results

### 3.1 Annual publication distribution

**Figure 1:**
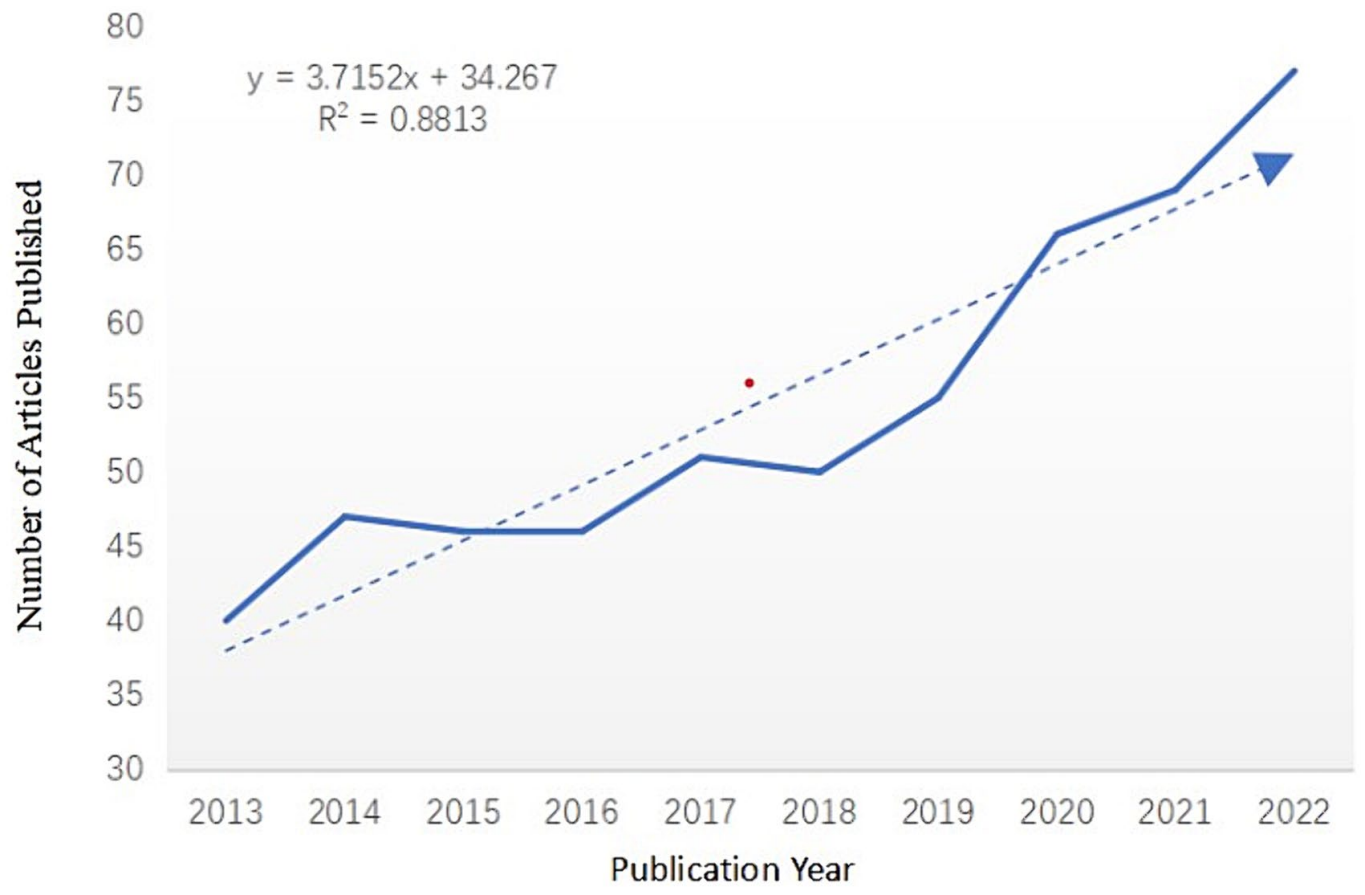
Annual number of publications on MCI influencing factors from 2013 to 2022.

This study analyzed 547 publications on factors affecting mild cognitive impairment in older adults published between 2013 and 2022. The citation report function of Web of Science was used to count the number of citations retrieved each year, and the citation data was independently verified using the repeat removal function of Cite Space software. Starting in 2019, the number of annual publications on this area of mild cognitive impairment is at a high level and on an upward trend, reaching a maximum in 2022. Figure 1 shows the number of publications per year for the last ten years.

### 3.2 Countries or regions

**Figure 2:**
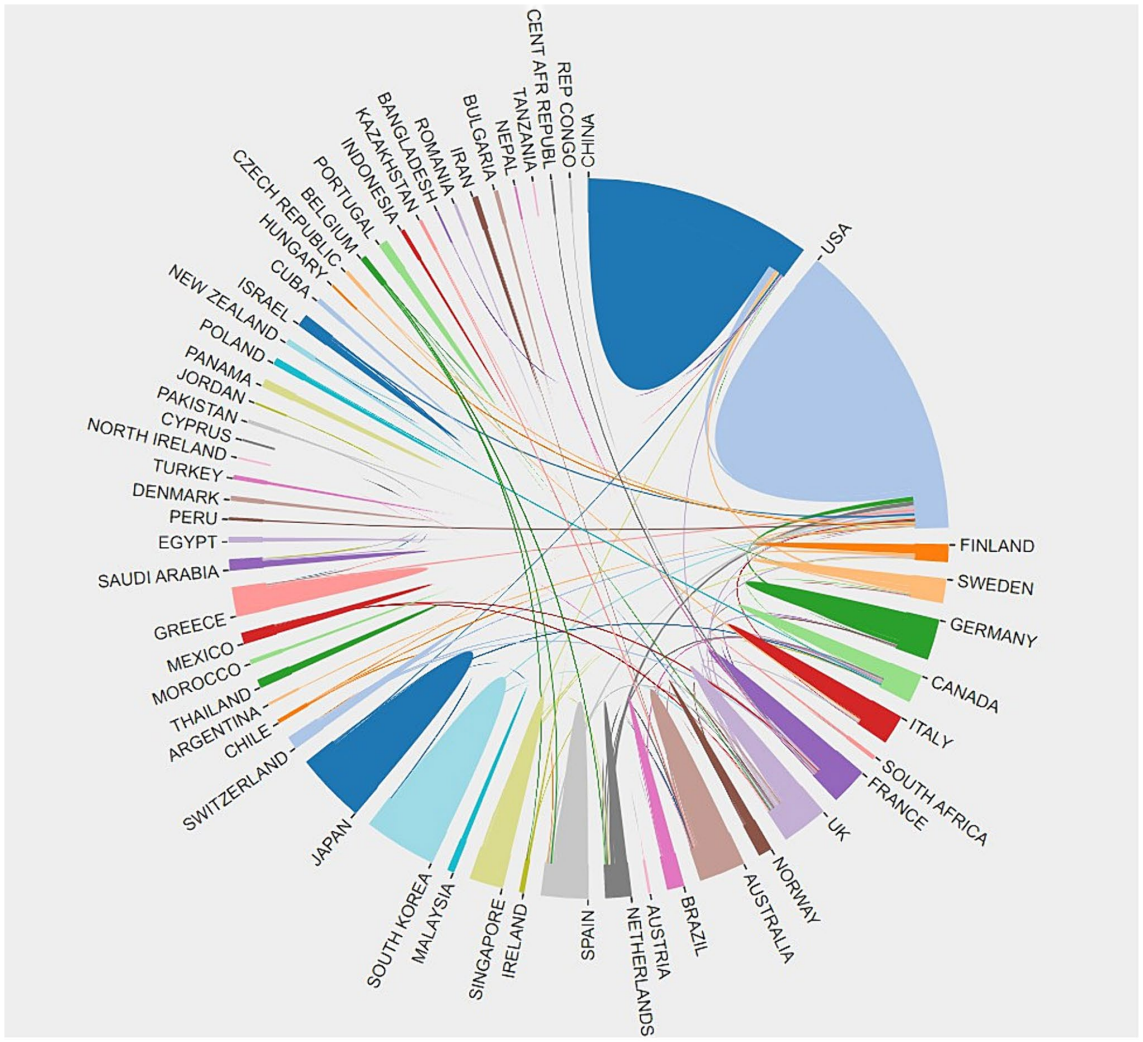
Cooperation among countries involved in the publication of MCI influencing factors, 2013–2022. Different colored areas represent various countries. The connection between different color blocks represents the countries’ cooperative relationship.

Retrieve published literature related to this field in all countries/regions, independently screen and discuss by two researchers, exclude literature unrelated to this field, and finally use bibliometric to analyze the publication volume and cooperation relationship of each country, with a total of 62 countries/regions were mentioned in the citations. The color-block areas in Figure 2 represent the proportion of publications by countries, and the different colored connecting lines represent the partnerships between countries. Compared to other color block areas, the United States, represented by light blue, and China, represented by dark blue, have the highest number of posts. The lighter blue areas have more connecting lines to the other color blocks, suggesting that the U.S. is working more frequently with other countries in the area of MCI impact factors. Table 1 shows the top five countries with the highest number of publications in the field of MCI impact factors, which are the United States (157), China (140), the United Kingdom (43), Japan (41), and South Korea (33). At present, research on the influencing factors of mild cognitive impairment mainly focuses on the United States and China. Some countries in Africa and South Asia, such as Tanzania, Morocco, Nepal, Kazakhstan, etc., have relatively few publications in this field and insufficient cooperation with other countries/regions. With the increasing international exchanges, the multi country cooperation model is gradually advancing.

**Table 1:** Top 5 countries with published papers on MCI influencing factors 2013–2022.

Rank	Country	Counts	Centrality	Percentage
1	America	157	0.34	28.70%
2	China	140	0.05	25.59%
3	England	43	0.31	7.86%
4	Japan	41	0.02	7.49%
5	Korea	33	0.01	6.03%

### 3.3 Institutional cooperation distribution

**Figure 3:**
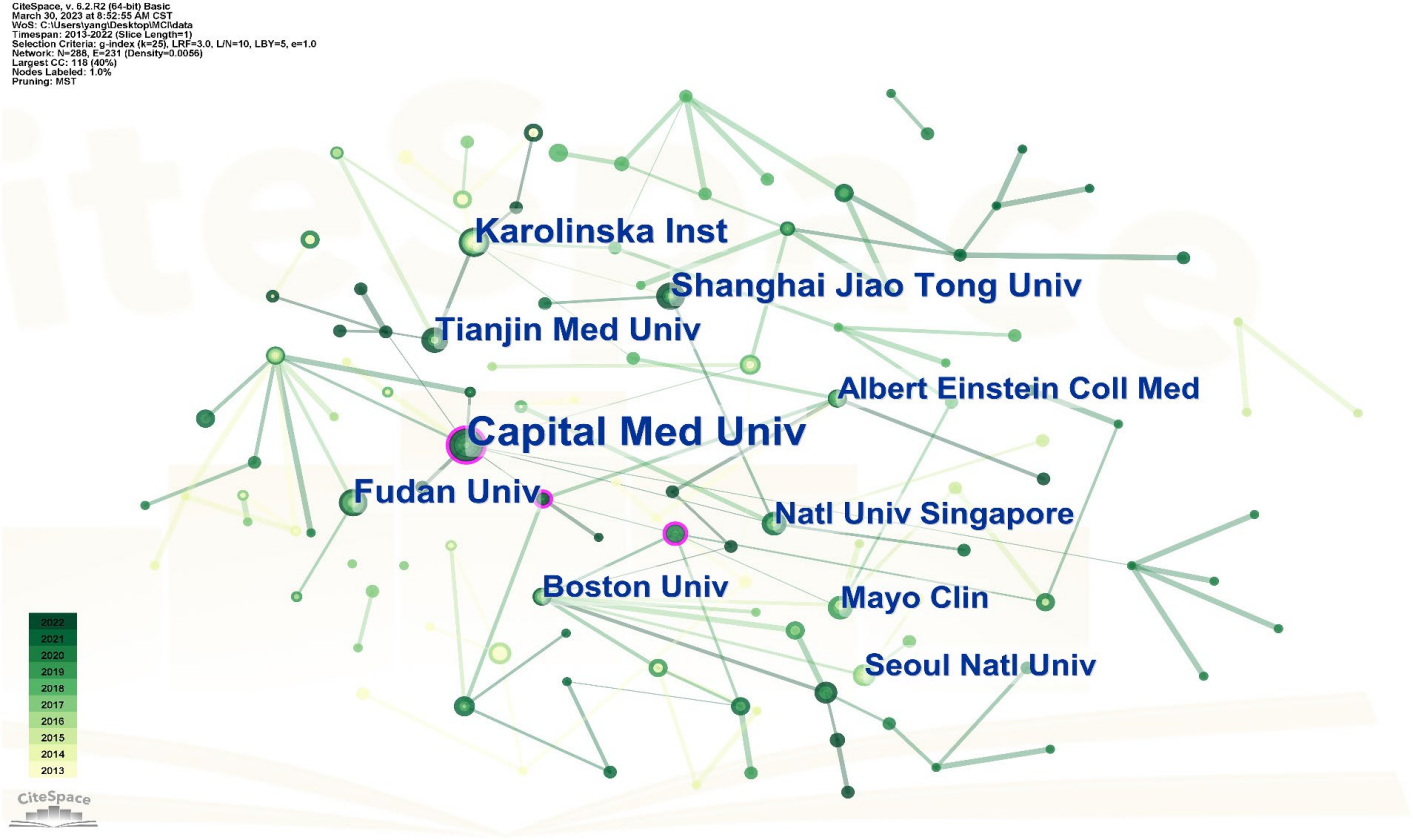
The chart of cooperation between institutions publishing papers in the field of MCI influencing factors from 2013 to 2022. Each label in the figure is a research institution. The connecting line between labels indicates the cooperative relationship between institutions.

Use Cite Space software to visualize and analyze the collaborative relationships and publication volumes between different institutions. As shown in Figure 3, a total of 288 organizations are involved in research in the area of MCI influencing factors during the period 2013–2022. This network graph generates a total of 288 nodes, 231 shortest paths of network nodes, each label represents an agency node, the connectivity between nodes represents the cooperation between agencies, and the size of the node represents the number of the number of messages sent by the agency. As shown in Table 2, the top ten institutions in terms of the number of publications in this field are Capital Medical University (18), Karolinska Institutet of Medicine (11), Shanghai Jiao Tong University (10), Fudan University (10), Tianjin Medical University (9), Boston University (8), Albert Einstein College of Medicine (8), Mayo Clinic (8), and National University of Singapore (8), Seoul National University (8 articles). In terms of institutional partnerships, Capital Medical University and Tianjin Medical University have more connecting lines with other nodes compared to other university institutions in the country, suggesting that the institution is working more closely with foreign institutions. However, relative to foreign countries such as Boston University and Mayo Clinic, China’s research in this field still needs to learn from foreign countries and strengthen exchanges and cooperation with foreign institutions to promote the development of this field.

**Table 2:** The top 10 institutions in the field of MCI influencing factors published in 2013–2022.

Rank	Institutions	Country	Counts	Centrality
1	Capital Medical University	China	18	0.19
2	Karolinska Institute	Sweden	11	0.05
3	Shanghai Jiao Tong University	China	10	0.02
4	Fudan University	China	10	0.01
5	Tianjin Medical University	China	9	0.01
6	Boston University	America	8	0.08
7	Albert Einstein College of Medicine	America	8	0.04
8	Mayo Clinic	America	8	0.02
9	National University of Singapore	Singapore	8	0.04
10	Seoul National University	Korea	8	0.01

### 3.4 Cited journals

**Figure 4:**
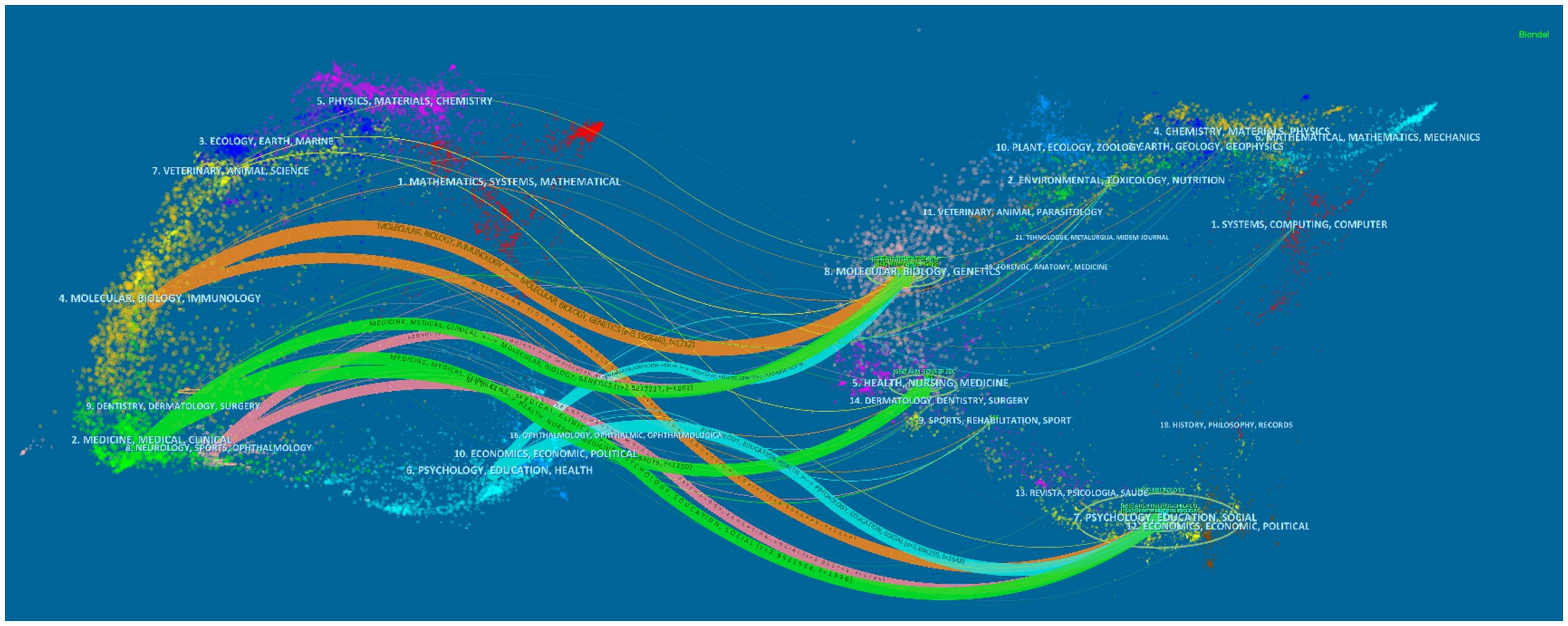
Double-graph overlay map of journals in the field of MCI influencing factors from 2013 to 2022. The left side correspond to the citation map and the right side represent the cited journal map. These labels represented the disciplines covered by the journal.

Through the analysis of the map of the cited journals published by the international MCI influencing factors from 2013 to 2022. As shown in Table 3, the most cited journal among the top 10 journals is NEUROLOGY with 439 citations in the last 10 years. The journal with the highest impact factor among the cited journals in the field is The Lancet, with an impact factor of 98.4 in 2024, which is of high quality and represents a high level of attention to the field of MCI impact factors. In order to have a more intuitive understanding of the relationship between the citing journal and the cited journal, this study uses Cite Space software to make the double-image overlay atlas of the journal. Figure 4 displayed the dual-map overlay of journals, the left and right sides corresponded to the citation map and the cited journal map, respectively. The color curve depicts the citations of journals from various disciplines. The arrow points to the cited publications from various disciplines that are typically referred to by citing journals. There were four citation paths. The yellow path shows articles in the research fields of MOLECULAR/BIOLOGY/IMMUNOLOGY that are more likely to cite articles in the field of GENETICS/EDUCATION/SOCIAL. The green path shows articles in the research fields of MEDICINE/MEDICAL/CLINICAL that are more likely to cite articles in the field of NURSING/PSYCHOLOGY/MOLECULAR. The pink path shows articles in the research fields of NEUROLOGY/SPORTS/OPHTHALMOLOGY that are more likely to cite articles in the field of BIOLOGY/PSYCHOLOGY/SOCIAL. The blue path shows the subject fields of BIOLOGY/GENETICS/SOCIAL, which are probably cited by PSYCHOLOGY/EDUCATION/HEALTH.

**Table 3:** Top 10 journals cited in the field of MCI influencing factors from 2013 to 2022.

Rank	Cited journals	Counts	2024 journal impact factor
1	NEUROLOGY	439	7.7
2	J ALZHEIMERS DIS	333	3.4
3	ALZHEIMERS DEMENT	318	13.0
4	J AM GERIATR SOC	316	4.3
5	DEMENT GERIATR COGN	243	2.2
6	INT J GERIATR PSYCH	213	3.6
7	LANCET NEUROL	212	46.5
8	PLOS ONE	211	2.9
9	LANCET	211	98.4
10	JAMA—J AM MED ASSOC	209	63.1

### 3.5 Analysis of keywords

**Figure 5:**
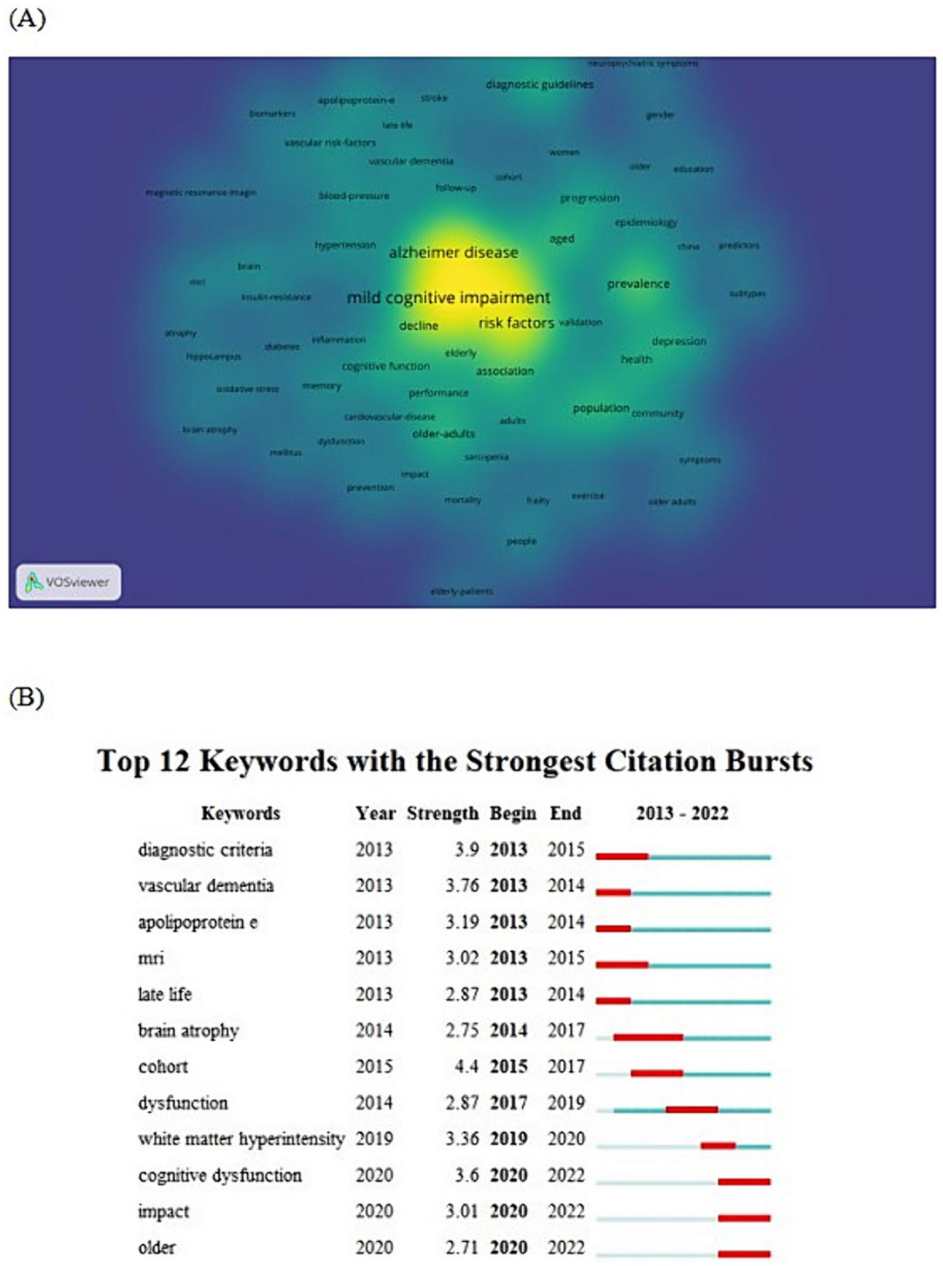
**(A)** Keywords popularity in literatures related to MCI influencing factors from 2013 to 2022. A keyword with higher frequency counts forms a yellow region, and those with lower frequency counts form a blue region. **(B)** Map of emerging keywords on MCI influencing factors from 2013 to 2022.

In this study, high frequency keywords were analyzed using Vos viewer software. To eliminate redundancy in the mapping, synonyms as well as abbreviated keywords were merged, such as Alzheimer-disease, Alzheimers-disease, AD, etc. into Alzheimer disease. The minimum threshold of keyword frequency was chosen as 11 and 80 keywords were visualized and analyzed. As shown in Figure 5A, the color of keyword labels with higher frequency of occurrence is closer to yellow, and the color of keyword labels with lower number of citations and frequency of occurrence is closer to blue. In research in the area of MCI influencing factors during the last 10 years, mild cognitive impairment, Alzheimers disease, dementia, risk factor, decline are high-frequency key words. Further Cite Space software was used to generate a table of emergent keywords, Figure 5B lists 12 emergent keywords from the timeline, observing the emergent words gives an idea of the high frequency keywords that appeared in different time-years, and thus the research hotspots in the field in each year.

### 3.6 Analysis of co-cited references

**Figure 6:**
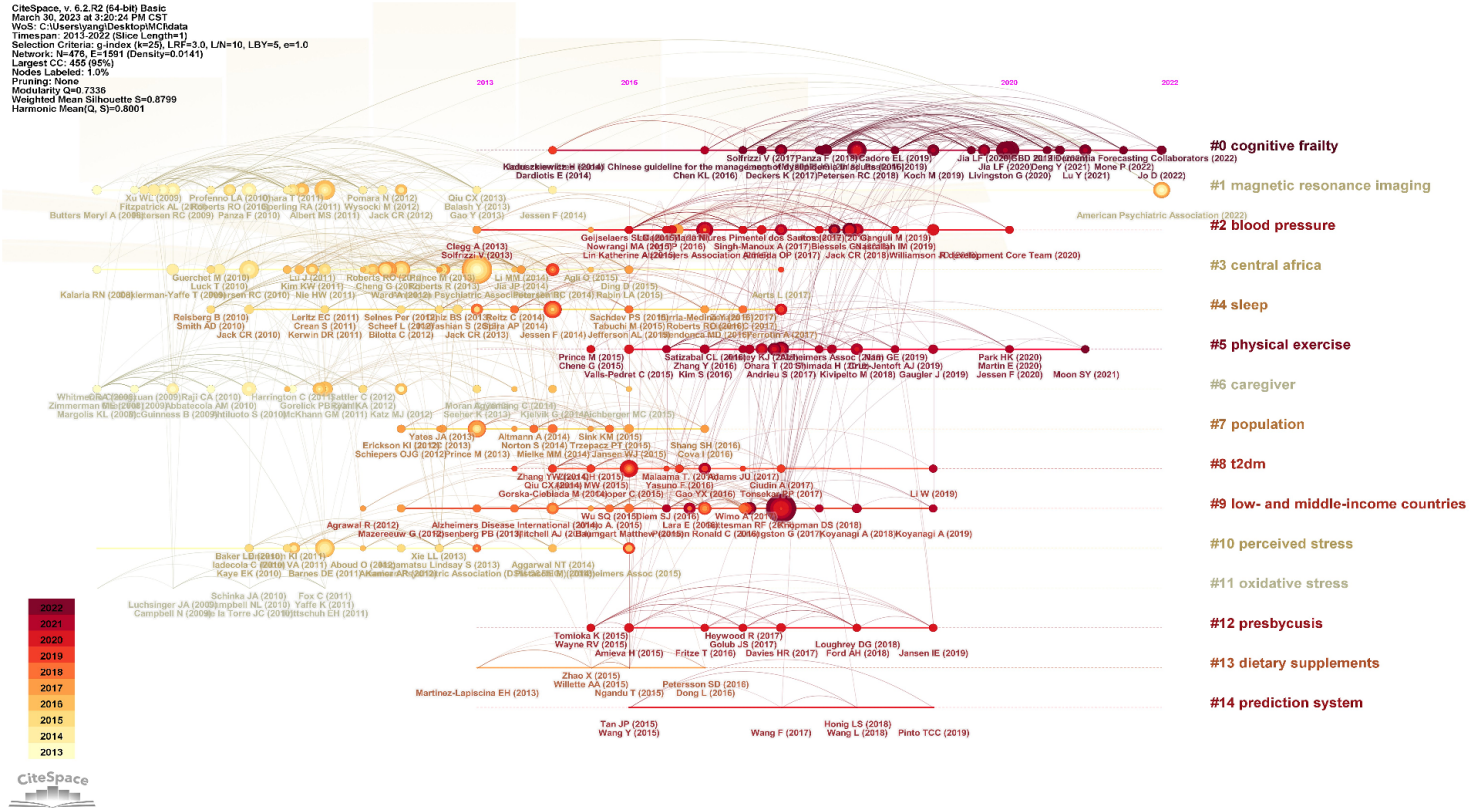
Co-citation time diagram of references on MCI influencing factors from 2013 to 2022. The clustering label of the co-cited literature may be found on the right side of the image.

By analyzing the co-citation timeline graphs of the literature, it is possible to understand the research themes and development of the research field in different time dimensions. The co-cited literature was clustered with keywords using the default algorithm in Cite Space, and a total of 15 clusters were generated, using the modularity index to measure the modularity of the network. The higher the modularity Q the better the clustering of the network. The graph clustering module value (Q-value) is 0.7336, the Q-value is more than 0.3, which implies that the clustering structure is significant; the average profile value (S-value) of the clusters is 0.8799, if the S-value is more than 0.5, it implies that the clustering is reasonable. As shown in Figure 6, the co-citation relationship of references on the timeline axis of different clustering groups changes with time and the citation heat, where the size of the nodes indicates the citation frequency of the literature. As can be seen in Figure 6, #cognitive frailty and #magnetic resonance imagine are important clusters and are hotly researched until 2022.

## 4 Discussion

### 4.1 Analysis of annual publications, countries, institutions, and journals

Articles on MCI’s influencing factors have increased dramatically since 2019. This may be related to the fact that more researchers are increasingly focusing on the field of geriatric MCI as the World Health Organization (WHO) officially releases guidelines for the prevention and control of cognitive decline and dementia risk in 2019. In the same year, Prof. Jia Jianping of Xuanwu Hospital of Capital Medical University published a study on the current situation of dementia patients in China in a Lancet sub issue (2). The proposed intervention programs and prevention and control strategies for dementia in China are of great significance in the prevention of dementia and cognitive impairment, and provide important scientific and technological references for researchers in the field of MCI. In terms of national cooperation, researchers in the United States and the United Kingdom are more closely involved with other countries in this area of cooperation and exchange. The U.S. has the most publications in this area and has the highest centrality of 0.34, and in Cite Space, a node with a centrality of more than 0.1 indicates that the node occurs more often on the shortest path in the overall network. The higher the centrality, the more important and influential the keyword is in the research area. Although China’s share of publications in this field is 25.59%, second only to the United States, its node centrality is only 0.05, and its influence in the field of MCI is relatively small, and it should strengthen cooperation and exchange with other countries to promote China’s research development in the field of MCI. In terms of institutional cooperation, Capital Medical University and Boston University have led the development of MCI in China and the United States, respectively, but the inter-institutional cooperation is in a small-scale and regionalized cooperation, while the international cooperation is less, and the institutions in China have not yet formed an obvious cooperation network in this field. Analyzing the research areas of the citing and cited journals, in the area of MCI influencing factors, more researchers are focusing on the cutting-edge areas of clinical medicine, neurology, immunology, and biology, which provide important scientific references for further dissecting the pathological changes of mild cognitive impairment and subsequent interventions.

### 4.2 Analysis of research hotspots

Keywords condense the core vocabulary of the subject matter of the literature and are a high level summary of the content of the literature, and high-frequency keywords are often used to identify hot issues in a field of study (14). Current research frontiers can be identified using emergent keyword analysis. Through the analysis of the keyword heat map generated by VOS viewer software and the co-citation time graph generated by cite space software, this study found that from 2013 to 2022, the research in the field of MCI influencing factors mainly included Alzheimer’s disease, risk factors, cognitive frailty and magnetic resonance imaging. The research focuses on the screening and effective prevention of cognitive function of the elderly in the community, so as to delay the development of MCI population towards AD.

#### 4.2.1 Cognitive frailty

Decrease in physiological reserve function of the elderly, leading to increased vulnerability of the body and causing debilitation of the elderly body (15). In contrast, cognitive debility is a subtype of debilitation with both physical debility and mild cognitive impairment. There are common risk factors for frailty and mild cognitive impairment, and frailty and mild cognitive impairment interact with each other (16, 17). Old age, low education, poor sleep quality and depression are common risk factors for frailty and mild cognitive impairment (18–20). There is a vicious cycle of interaction between mild cognitive impairment and frailty, which makes older adults with organic frailty more susceptible to mild cognitive impairment. Shimada H’s study explored the relationship between cognitive decline and the prevalence of dementia in community-dwelling older adults and found that community-dwelling older adults with cognitive decline had a higher prevalence of dementia than older adults with mild cognitive impairment alone or older adults with debility (21). Frail older adults have reduced visuospatial ability, executive ability, and lower delayed memory scores compared to older adults in the normal community (22). The common risk factors and high-risk groups of mild cognitive impairment and frailty are gradually clear, but there is a lack of large-scale experimental studies, and the relevant mechanism of frailty leading to cognitive changes in various dimensions still needs to be further explored. In conclusion, healthcare professionals should pay high attention to the status of organic weakness and cognition in the elderly in order to recognize and prevent cognitive weakness in the elderly at an early stage.

#### 4.2.2 Alzheimer’s disease

Alzheimer’s disease is one of the most common forms of dementia, severely affecting the central nervous system of the brain, resulting in cognitive dysfunction and mental behavior abnormalities (23). After a diagnosis of mild cognitive impairment, most older adults with mild cognitive impairment will transition to dementia within 4–6 years without intervention (24). At present, most studies focus on the influential factors of mild cognitive impairment and Alzheimer’s disease in the elderly, and few studies have explored the influential factors of the transformation of patients with mild cognitive impairment into Alzheimer’s disease. The Berezuk researchers investigated the risk factors for the conversion of MCI to AD, and the results showed that advanced age, female gender, hypertension, diabetes, residential status, neuropsychiatric symptoms, and cerebrovascular disease were risk factors for the conversion of MCI patients to AD (25–27). Kewcharoen’s study found that patients older than 75 years of age with MCI were more likely to develop AD (28). Udeh-Momoh C’s study found that in the elderly population with MCI, women are more likely to develop AD than men, which may be related to the greater changes in estrogen levels in women in old age (29, 30). Neuropsychiatric symptoms are one of the risk factors for the conversion of MCI to AD, which may be related to the development of negative emotions (31), and depression is the most common symptom of MCI progression to AD (32). Through the graph analysis of the cited journals in the international MCI research hotspots in the past decade, it is found that the literatures in the fields of psychology and nursing are more frequently cited in medical and clinical journals. More and more researchers pay attention to the neuropsychiatric symptoms of MCI and improve the cognitive function of patients through psychological intervention. Analysis of risk factors for the development of AD patients in older adults with mild cognitive impairment, early screening and individualized clinical management of patients with mild cognitive impairment are beneficial for improving cognitive function and reducing the incidence of MCI to AD (33, 34).

#### 4.2.3 Magnetic resonance imaging

Neuroimaging techniques provide an important diagnostic basis for exploring structural and functional changes in the brain of older adults with mild cognitive impairment, and play an important role in the early diagnosis and risk prediction of mild cognitive impairment (35). Amnestic mild cognitive impairment is mainly characterized by memory loss (36), which is closely related to hippocampal atrophy (37). Amnestic mild cognitive impairment declines more rapidly relative to other subtypes of cognitive functioning and progresses more rapidly to AD (38). The Montreal Cognitive Assessment Scale is a widely used instrument for assessing cognitive functioning in older adults, with good levels of content and structural validity (39). Through MRI, Knudsen LV found that brain structural abnormalities in patients with mild cognitive impairment have a higher correlation with clinical test scores, and MRI can more accurately identify different types of MCI, which is convenient for early intervention and treatment of MCI in elderly people (40). Resting-state functional MRI has the advantages of being noninvasive, high spatial resolution, and comparability. Cai investigated the mechanism of progression of aMCI to AD by using MRI and found that patients with aMCI have diminished functional connectivity in default network brain regions such as the hippocampus, medial prefrontal and inferior parietal lobes (41). MRI is widely used to detect and understand the development of neurodegenerative diseases such as MCI, with high detection accuracy and generalization ability, and it is possible to differentiate between stable and progressive mild cognitive impairment by analyzing magnetic resonance imaging images (42). In observing the extent of volume changes in hippocampal subregions and the functional connectivity characteristics among subregions, MRI can provide more detailed and accurate reference value for MCI diagnosis and disease progression.

## 5 Limitations

There are limitations to this visualization analysis, which currently only includes literature published in the last decade from 2013–2022; some studies are ongoing but not yet published. Secondly, the analysis of this study only included literature data in Web of Science and lacked analysis of different databases such as PubMed and Cochrane. Subsequent studies can add databases to expand the scope of searching, while supplementing Chinese databases to compare and analyze domestic and foreign studies, identify differences, and analyze them in depth.

## 6 Conclusion

This study objectively analyzes the research hotspots and research frontiers in the field of MCI influencing factors in older adults through the use of bibliometric methods. The results show that the research focus in this field is mainly on Alzheimer’s disease and risk factors in recent ten years. The clinical therapeutic medications for AD can only alleviate psychological and behavioral symptoms to a certain extent, and cannot prevent the progression of AD. Therefore, more and more researchers’ attention is focused on MCI, and understanding the risk factors that impair cognitive functioning during this period is beneficial in slowing down the progression of MCI to AD. In addition, cognitive decline and MRI are at the forefront of research in the MCI field, and community healthcare workers should focus on older adults with decline and screen for cognitive function at an early stage.
